# Correction: Division of Labor in Hand Usage Is Associated with Higher Hand Performance in Free-Ranging Bonnet Macaques, *Macaca radiata*

**DOI:** 10.1371/journal.pone.0126902

**Published:** 2015-04-20

**Authors:** The PLOS ONE Staff

## Notice of Republication

This article was republished on April 8, 2015, to correct an error in the title and abstract that was introduced during the production process: "*Macaca radiata*" was changed to "*Macaca radiate*.” The publisher apologizes for the error. Please download this article again to view the correct version. The originally published, uncorrected article and the republished, corrected article are provided here for reference.

## Supporting Information

S1 File Originally published, uncorrected article. (PDF)

S2 File Republished, corrected article. (PDF)

## References

[1] Mangalam M, Desai N, Singh M (2015) Division of Labor in Hand Usage Is Associated with Higher Hand Performance in Free-Ranging Bonnet Macaques, *Macaca radiata* . PLoS ONE 10(3): e0119337 doi:10.1371/journal.pone.0119337 25806511 10.1371/journal.pone.0119337 PMC4373831

